# Temperature Dependence of Photodegradation of Dissolved Organic Matter to Dissolved Inorganic Carbon and Particulate Organic Carbon

**DOI:** 10.1371/journal.pone.0128884

**Published:** 2015-06-24

**Authors:** Petr Porcal, Peter J. Dillon, Lewis A. Molot

**Affiliations:** 1 Environmental and Resource Studies, Trent University, Peterborough, Ontario, Canada; 2 Biology Centre of the Czech Academy of Sciences, v.v.i., Institute of Hydrobiology, České Budějovice, Czech Republic; 3 Faculty of Environmental Studies, York University, Toronto, Ontario, Canada; University of Aveiro, PORTUGAL

## Abstract

Photochemical transformation of dissolved organic matter (DOM) has been studied for more than two decades. Usually, laboratory or “in-situ” experiments are used to determine photodegradation variables. A common problem with these experiments is that the photodegradation experiments are done at higher than ambient temperature. Five laboratory experiments were done to determine the effect of temperature on photochemical degradation of DOM. Experimental results showed strong dependence of photodegradation on temperature. Mathematical modeling of processes revealed that two different pathways engaged in photochemical transformation of DOM to dissolved inorganic carbon (DIC) strongly depend on temperature. Direct oxidation of DOM to DIC dominated at low temperatures while conversion of DOM to intermediate particulate organic carbon (POC) prior to oxidation to DIC dominated at high temperatures. It is necessary to consider this strong dependence when the results of laboratory experiments are interpreted in regard to natural processes. Photodegradation experiments done at higher than ambient temperature will necessitate correction of rate constants.

## Introduction

Dissolved organic matter (DOM) is a significant source of energy and nutrients in the food chain [1] and photochemical reactions plays a major role in transforming DOM and releasing its bound nutrients. Photochemical transformation of DOM changes its inner structure [2], produces lower molecular weight fragments [3], and undergoes complete oxidation to CO and CO_2_ [4, 5]. Moreover, photochemical transformation of DOM results in the loss of color and the concentration of organic carbon. The photochemically induced changes can increase or decrease DOM bioavailability [6] and enhance microbial decomposition [7].

Usually, laboratory or “in-situ” experiments are used to determine photodegradation variables. In-situ experiments expose samples to natural solar radiation in its own environment [8], while pseudo in-situ experiments use only natural solar radiation under different conditions e.g. [9, 10]. Laboratory experiments, however, enable precise control of irradiation conditions. Some experiments use whole spectrum solar light simulators e.g. [11, 12], while others use a particular range of wavelengths e.g. [13, 14]. The interpretation and interpolation of results to natural conditions is difficult. The problem with the use of different irradiation sources and intensities during different experiments can be eliminated by expressing the results as a function of total irradiation energy exposure [12]. Another common problem with these experiments is that the temperature at which they are conducted differs from the ambient temperature. Molot and Dillon [9] in surface exposure experiments compared photodegradation rate constants with corresponding mass balance rates and assumed that the reaction rates changed twofold with each 10°C change, implicitly assuming that the relative importance of the various pathways did not change with temperature.

This study was aimed at determining the effect of temperature on photochemical degradation of DOM. Laboratory experiments with different temperature conditions were conducted and the results modeled to describe the effect of temperature on photochemical degradation pathways.

## Material and Methods

Water used in this study was collected from a long term monitoring site (Plastic 1 tributary to Plastic Lake, Dorset region, Ontario, Canada; 45.1793° N, 78.8280° W) in May 2009. The study site is near the southern edge of Precambrian Shield and the boundary of the Boreal ecozone. Detailed description and characterization of the study site and its catchment is in Dillon et al. [15]. This site has been used in previous photochemistry experiments [9, 16, 17, 18].

Sample water was collected in 20 liter PET bottles, previously acid washed and thoroughly rinsed with demineralized water and sample. Samples were filtered through a series of polypropylene cartridge filters with decreasing pore size (10–0.5 μm; U.S. Filters) and stored in the dark at 4°C. The possible effect of storing for 6 weeks, tested by comparing the results of photochemical experiments done with the same stored water under the same conditions (irradiation intensity of 700 W m^-2^; room temperature; duration 48 hours) before and after temperature experiments, revealed no significant differences in TOC concentration (decrease of 25%) and absorbance spectrum (e.g., decrease in absorbance at 280 nm of 39%; measured in 1 cm cuvette, Cary 50 UV-Vis spectrophotometer, Varian) (paired t-test; t = 0.66; df = 3; p > 0.05). Basic chemical parameters of the water sample are presented in Table 1.

**Table 1:** Initial composition of filtered sample.

Parameter	Units	Concentration
**pH**		4.7 ± 0.04
**Na^+^**	(mg L^-1^)	0.42 ± 0.01
**K^+^**	(mg L^-1^)	0.11 ± 0.02
**Ca^2+^**	(mg L^-1^)	0.66 ± 0.01
**Mg^2+^**	(mg L^-1^)	0.19 ± 0.01
**NH_4_^+^**	(μg L^-1^)	18 ± 2
**Cl^-^**	(mg L^-1^)	0.02 ± 0.02
**SO_4_^2-^**	(mg L^-1^)	1.75 ± 0.08
**NO_3_^-^**	(μg L^-1^)	10 ± 0.2
**alkalinity**	(mg L^-1^ as CaCO_3_)	-1.3 ± 0.1
**Al**	(μg L^-1^)	288 ± 8
**Fe**	(μg L^-1^)	294 ± 16

Aluminum and iron were determined by ICP-MS (Thermo X II ICPMS). Other analytical methods are described in Ontario Ministry of the Environment [19].

**Fig 1:**
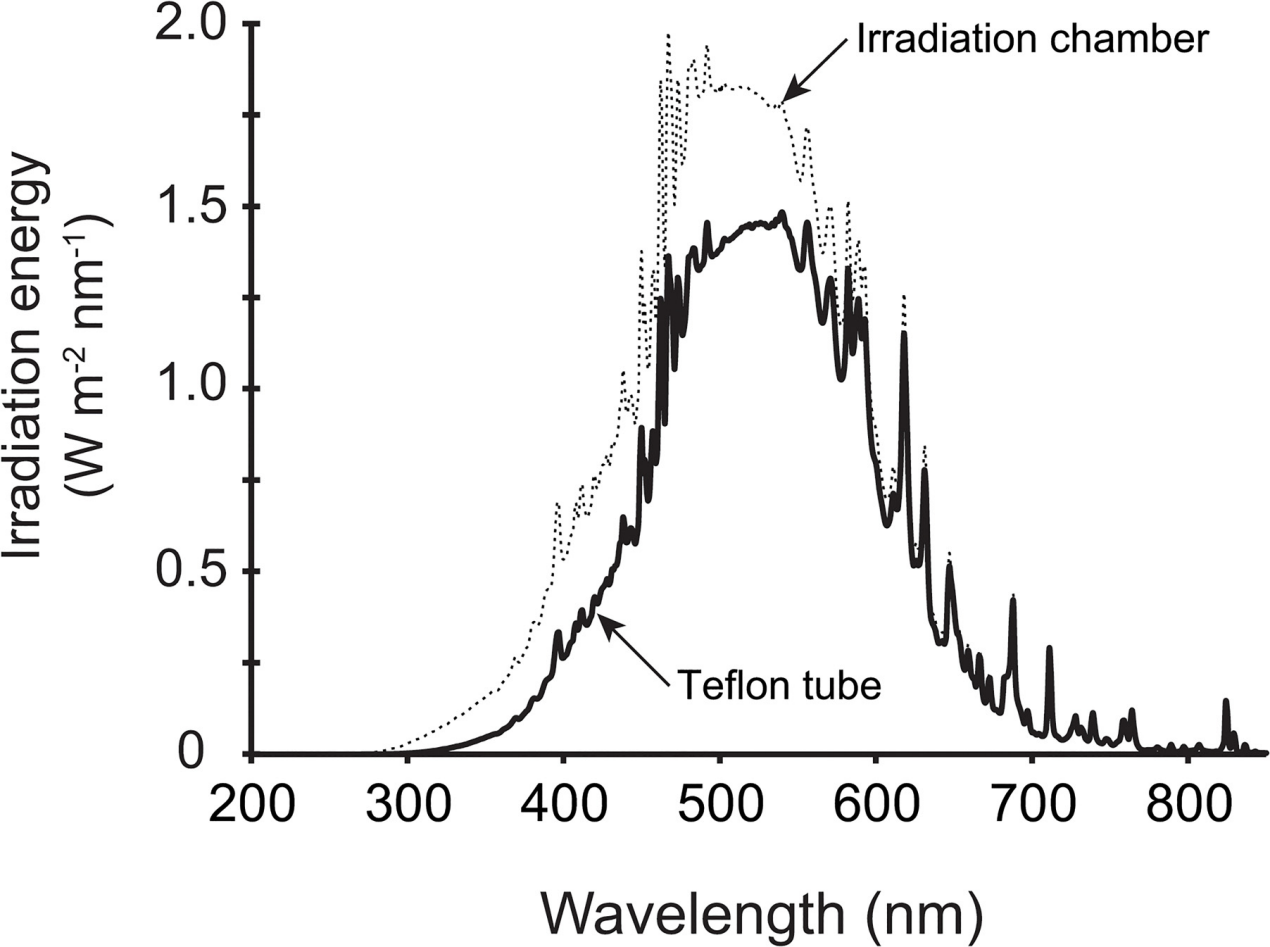
Spectral distribution of irradiation at intensity 700 W m^-2^ inside the irradiation chamber and inside the Teflon tube.

Before each experiment the sample was vacuum filtered through the 0.45 μm cellulose acetate membrane filter (Nalgene). Irradiation experiments took place inside an irradiation chamber (Suntest XLS+, Atlas, Germany) equipped with a Xenon lamp simulating solar radiation at sea level with an intensity of 700 W m^-2^ (Fig 1). Samples were irradiated in a Teflon tube exposed inside the irradiation chamber. The inner diameter of Teflon tube was 10 mm and the thickness of its wall was 1 mm. The Teflon tube was coiled in one layer on the bottom of the irradiation chamber, and the distance between adjoining coils was 5 mm. The inner volume of Teflon tube exposed to irradiation was 200 mL. The apparatus was washed by a detergent (Sparkleen, Fisher Scientific), followed by 1M HCl and 1M NaOH and thoroughly rinsed with de-mineralized water before each experiment.

The spectrum of light transmitted through the Teflon wall is presented in Fig 1. The shading effect of Teflon tube can be expressed as an attenuation depth. The attenuation depth was calculated as the ratio of percent attenuation depth and attenuation coefficient [20]. Percent attenuation depth corresponded to a 60% decrease in UV radiation at 380 nm. The attenuation coefficient of our sample estimated according to a model (K_D_ = exp(-0.01347·λ) + 5.36·DOC^0.157^ for λ = 380 nm and DOC = 12.8 mg L^-1^) was 16 m^-1^ [21]. Attenuation depth corresponding to thickness of Teflon tube was thus equal to 3 cm of sample. Hence, laboratory experiments were representative of photochemical processes in the surface layer of water bodies.

The irradiation chamber was cooled by air blown in from an attached air condition unit. The intensity of cooling was driven by a thermostat inside the irradiation chamber set to 25°C. It was impossible to cool the whole irradiation unit to lower temperature because of intense heat emitted from the source lamp. Thus, the entire sample (2.5 L) was not irradiated at once, but was stored in a PET bottle placed in a cooler filled with ice to keep its temperature as low as possible and continuously peristaltically pumped through the Teflon tube in the irradiation chamber using a silicone tube inside the pump head. Different temperature regimes were established by changing the ratio between air, water and ice inside the cooler. The flow through the Teflon tube was set to 250 mL min^-1^. All tubing outside the irradiation chamber was insulated and wrapped in a tin foil. The ice was continuously replenished. The ice was substituted with air during experiments at lab temperature. The temperature of the sample was recorded using a temperature data logger (HOBO Water Temp Pro, HOBO, U.S.A.) submerged in the PET bottle. The mean temperature was chosen to represent the temperature of each experiment and ranged from 9 to 25°C in all experiments. Duration of each experiment was 5 days.

Sub-samples were collected six times during the experiment and the concentrations of total and dissolved organic carbon (TOC and DOC) were determined in sub-samples. The volumes of collected sub-samples and final volume of sample in the system were recorded to determine possible evaporation or leakage. Samples for DOC analysis were filtered through 0.45μm acetate cellulose filter (Nalgene) to remove possibly formed particles [17, 22]. Duplicate sub-samples were measured. TOC and DOC were determined with a TOC-VC_P_ analyzer (Shimadzu, Japan) by catalytic combustion oxidation with non-dispersive infrared detection. TOC and DOC contents were calculated as the difference between total carbon (TC) and inorganic carbon (IC) measured after sample acidification with phosphoric acid (25% by weight diluted from ≥85 wt. % in H_2_O, Sigma-Aldrich, USA). The content of IC in all samples did not exceed 5% of TC. Inorganic and organic standards (sodium carbonate monohydrate, ≥99.5%; sodium carbonate, anhydrous, ≥99.5%; potassium hydrogen phthalate, p.a., ≥99.5%, Sigma-Aldrich, USA) were used for instrument calibration as well as external standards and were analyzed at the beginning and at the end of each run to correct for possible instrumental drift.

The concentration of particulate organic carbon (POC) was calculated as the difference between TOC and DOC concentrations. The term particulate used in this study refers to particles larger than 0.45 μm. TOC and DOC concentrations in irradiated samples determined in duplicates were statistically significantly different in all sample pairs (paired t-test; t = 5.74; df = 19; p<0.001). Concentrations of TOC and DOC in initial non-irradiated samples, filtered through the 0.45μm filter prior the experiment, were not significantly different (paired t-test; t = 0.15; df = 3; p > 0.05). Control samples wrapped in a tin foil, occasionally mixed and stored at the same temperature as irradiated samples were measured during each experiment to determine possible changes in TOC and DOC concentrations due to microbial activity. Comparison of control samples continuously pumped to control samples without pumping did not reveal any significant difference between pumped and non-pumped samples for the duration of experiment (t-test; t = -1.7; df = 5; p > 0.05). The concentrations of TOC in control samples were similar to the concentration at the beginning of experiments (t-test; t = 2.4; df = 6; p > 0.05). The concentrations of DOC in control samples were similar at lower temperatures, however, at temperatures 24° and 25°C, the concentration of DOC in control samples was 3 ± 0.8% lower at the end of the experiment than at the beginning of irradiation (t-test; t = 6.6; df = 3; p < 0.05), hence, results of irradiated samples were corrected for these differences. The changes in control samples were linearly extrapolated for each individual time period and these changes, which were attributed to processes other than irradiation, were added to the measured DOC concentrations at a given time. Based on organic carbon mass balance, the concentration of dissolved inorganic carbon (DIC) was calculated as the difference between initial TOC concentration and TOC concentration at any time.

Experiments were conducted with subsamples under the same irradiation conditions to enable better comparison of temperature effects. The cumulative energy of irradiation exposure was 33.6 MJ m^-2^. The intensity of irradiation was 700 W m^-2^. It is approximately two times higher than monthly averaged midday insolation on horizontal surface in June at 44° N, 78° W and approximately 30% higher than maximum outdoors irradiance under a cloudless, non-hazy sky on June 21. The higher intensity was used to reduce irradiation time to accelerate the experimental work. Preliminary experiments, with samples with DOC concentration ranging from 0.4 to 3.3 mmol L^-1^ irradiated under two intensities (400 and 700 W m^-2^) for the same dose of irradiation energy, confirmed that the photodegradation rate constant does not depend on intensity of irradiation, but on the total amount of irradiation energy exposure (paired t-test, p>0.05, n = 4) [18].

Radiation incident on a horizontal surface (global horizontal radiation) is a common meteorological parameter which can be used to compare the results of laboratory experiments under natural conditions. Monthly averaged insolation incident on a horizontal surface for our sampling location were used to compare energy of natural and artificial irradiation (NASA Surface meteorology and Solar Energy, http://eosweb.larc.nasa.gov). Global horizontal radiation is measured over a wide spectrum range including UV, visible and near-infrared radiation. However, the radiation inside of the irradiation chamber was only measured within UV and visible spectrum range. Global horizontal radiation had to be corrected. The portion of near-infrared radiation in global horizontal radiation ranges from 46 to 52% e.g. [23, 24] and references therein, depending on atmospheric conditions, e.g. amount of water vapor. In our calculations the global horizontal radiation data were reduced by 50% to eliminate the portion of near infrared radiation. Comparison of natural and artificial irradiation showed that the cumulative energy of irradiation per experiment 33.6 MJ m^-2^ corresponded to 2.3 days in June with a clear, no-hazy sky at our sampling location.

The initial concentrations of Al, and Fe in dissolved forms were determined by inductively coupled plasma mass spectroscopy with collision cell technology (Thermo X II ICPMS, USA). Other initial chemical parameters (Table 1) were determined according to Ontario Ministry of the Environment [19].

### Mechanism of photochemical transformation of DOM

Photochemical transformations of DOM are described by changes in three carbon fractions, DOC, POC and DIC, which can be easily measured or calculated.

**Fig 2:**
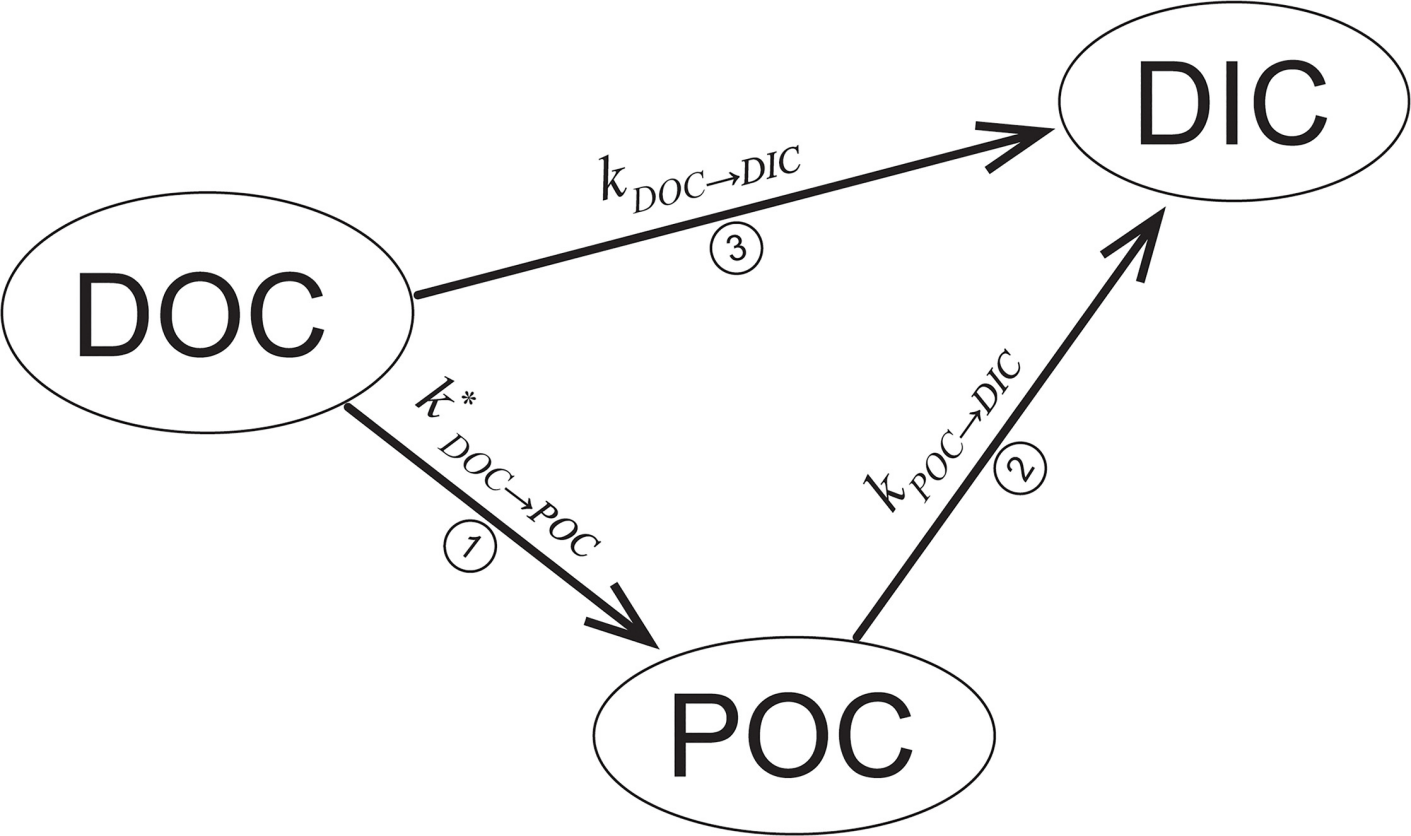
Scheme of possible transformations of organic matter during exposure to irradiation. DOC–dissolved organic carbon, POC–particulate organic carbon, DIC–dissolved inorganic carbon; *k*_*DOC→DIC*_, k*_DOC→POC_, and *k*_*POC→DIC*_ are rate constants of corresponding transformation pathways.

Mechanisms accounting for photochemical transformation of DOM are presented in Fig 2. The concentration of DOC is decreased by its photochemical transformation to POC, transformation pathway 1 in Fig 2 [17], and by oxidation to DIC, transformation pathway 3 in Fig 2 [5]. The complex mechanism of DOC changes is described by Eq 1:

$$\frac{dDOC}{dt}=-(k_{DOC\to POC}^{*}+k_{DOC\to DIC})\left[DOC\right],$$ (1)

where k*_DOC→POC_ is the equilibrium rate constant combining rate constants of POC production and possible POC disaggregation which increases the DOC concentration, and *k*_*DOC→DIC*_ is the rate constant for DIC production from DOC.

The formation of POC followed by its photochemical oxidation to DIC, transformation pathway 2 in Fig 2, is described by Eq 2:

$$\frac{dPOC}{dt}=k_{DOC\to POC}^{*}\left[DOC\right]-k_{POC\to DIC}\left[POC\right],$$ (2)

where *k*_*POC→DIC*_ is the rate constant of POC photochemical oxidation to DIC.

Thus DIC is formed by two photochemical oxidation processes and is described by Eq 3:

$$\frac{dDIC}{dt}=k_{DOC\to DIC}\left[DOC\right]+k_{POC\to DIC}\left[POC\right],$$ (3)

where *k*_*DOC→DIC*_ and *k*_*POC→DIC*_ are rate constants of DIC production from DOC and POC, respectively.

The mass balance of carbon in DOC, POC and DIC fractions was calculated by solving the system of equations (Eqs 1–3). For the solution of these differential equations we assumed pseudo first order kinetics for all processes, because the mechanism has not been specifically identified, but the observed changes were consistent with a first order kinetics [25].

The changes in DOC, POC, and DIC are expressed against exposure time, *t*, which is calculated according to Eq 4:

$$t=D\cdot \frac{V_{e}}{V},$$ (4)

where *D* is the duration of experiment (h), *V*_*e*_ is the volume of exposed Teflon tube inside the irradiation chamber (200 ml), *V* is the total volume of sample in the system.

The concentration of POC at any time is calculated as:

$$POC=\frac{k_{DOC\to POC}^{*}}{K}DOC_{0}\frac{\left(e^{K\cdot t}-1\right)}{e^{k_{POC\to DIC}\cdot t}},$$ (5)

where *t* is the exposure time (h), *DOC*_*0*_ is the initial DOC concentration and *K = k*_*POC→DIC*_ *− k*_*DOC→POC*_ *− k*_*DOC→DIC*_, where *k*_*DOC→POC*_, *k*_*POC→DIC*_, and *k*_*DOC→DIC*_ are pseudo first-order kinetic rate constants (h^-1^) for the transformation pathways (Fig 2).

DIC produced from POC (*DIC*_*POC*_) was calculated according to the Eq (6):

$$DIC_{POC}=\frac{k_{DOC\to POC}^{*}}{\left(k_{DOC\to DIC}+k_{DOC\to POC}^{*}\right)}\cdot DOC_{0}\cdot (1-e^{-(k_{DOC\to POC}^{*}+k_{DOC\to DIC}^{})\cdot t})-POC,$$ (6)

where *POC* was calculated according to the Eq 5.

DIC photochemically produced from DOC (*DIC*_*DOC*_) was calculated as follows:

$$DIC_{DOC}=\frac{k_{DOC\to DIC}}{\left(k_{DOC\to DIC}+k_{DOC\to POC}^{*}\right)}\cdot DOC_{0}\cdot (1-e^{-(k_{DOC\to POC}^{*}+k_{DOC\to DIC})\cdot t}).$$ (7)

The non-linear least square regression method was used to fit measured data with the system of equations (Eqs 5–7) to determine the kinetic rate constants of individual transformation processes. The coefficient of determination (R^2^) was calculated as:

$$R^{2}=1-\frac{\sum {\left(y-y_{fit}\right)}^{2}}{\sum {\left(y-y_{mean}\right)}^{2}}$$ (8)

where *y* represents measured data, *y*_*fit*_ is modeled data, and *y*_*mean*_ is the mean of measured data. Uncertainty propagation for modeled parameters was determined by fitting modeled data with a 95% confidence interval, which was calculated as:

$$CI=\sqrt{\frac{\sum {\left(y-y_{fit}\right)}^{2}}{n-p}\cdot t^{*}}$$ (9)

Where *n* represents degrees of freedom, *p* is a number of parameters determined during one regression and t* is the critical value for the t distribution with *n* – 1 degrees of freedom. All calculations were done in Microsoft Excel by the Solver Add-In. Statistics were calculated with Statistica 8.0 software (www.statsoft.com).

### Permissions

Water samples from headwater stream (Plastic 1) were collected under the auspices of pre-existing Ontario Ministry of the Environment research program. Specific permissions for these samplings were not required. No endangered or protected species were involved.

## Results and Discussion

Five laboratory experiments were done between 9 and 25°C to determine the effect of temperature on photochemical degradation of DOM. Two additional experiments were done under the same conditions at laboratory (i.e., room) temperature (approximately 23°C), but the temperatures in these experiments were not continuously recorded. Regardless, their results are presented in Table 2 for comparison with experiments with continuously recorded temperature.

**Table 2:** Modeled initial concentrations, and individual transformation pathway rate constants at different temperatures ± standard errors.

Mean temperature	DOC_0_	*k*_*DOC→DIC*_	k*_DOC→POC_	*k*_*POC→DIC*_	R^2^
(°C)	(mg L^-1^)	(h^-1^)	(h^-1^)	(h^-1^)	
9°C	12.7 ± 0.6	0.033 ± 0.001	0.002 ± 0.0011	0.002 ± 0.001	0.996
10°C	12.8 ± 0.6	0.035 ± 0.0013	0.003 ± 0.0013	0.003 ± 0.0009	0.996
14°C	12.7 ± 0.7	0.011 ± 0.0091	0.034 ± 0.0034	0.076 ± 0.0212	0.962
24°C	12.6 ± 0.7	0 ± 0.0028	0.06 ± 0.0019	0.264 ± 0.0144	0.992
25°C	12.7 ± 0.6	0 ± 0.0004	0.064 ± 0.0014	0.324 ± 0.0022	0.995
L.T.	12.4 ± 0.8	0 ± 0.0155	0.057 ± 0.0121	0.189 ± 0.0256	0.982
L.T.	12.5 ± 0.8	0 ± 0.0167	0.058 ± 0.0132	0.152 ± 0.0385	0.982

L.T.–laboratory temperature, approximately 23°C.

**Fig 3:**
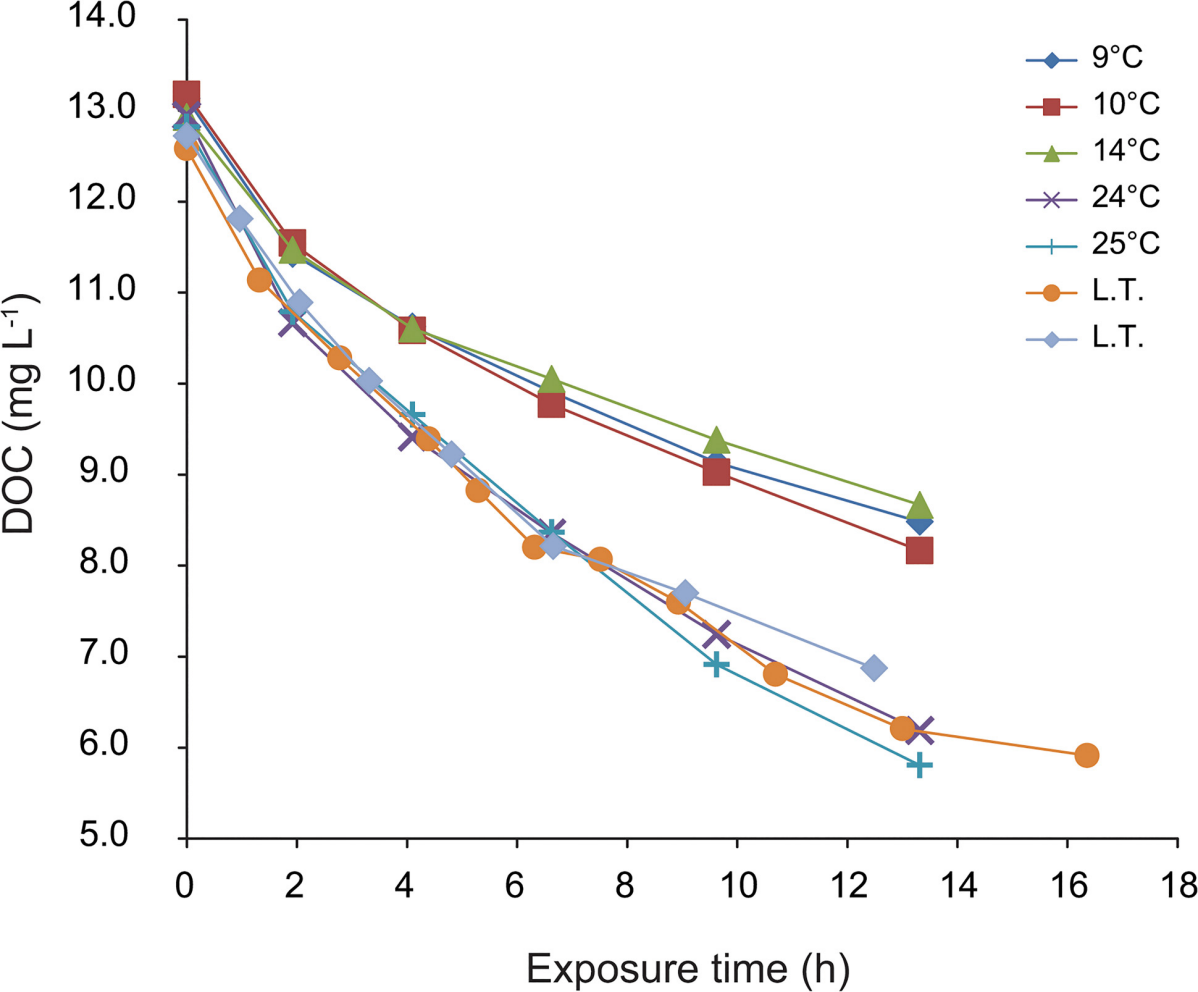
Changes in DOC concentration during irradiation experiments at different temperatures. L.T.–laboratory temperature, approximately 23°C.

Concentrations of TOC and DOC decreased during irradiation in all experiments. The largest decrease in TOC concentration of 49% of the initial value was observed at 24°C. Decreases in TOC concentration were similar at temperatures 9°C and 14°C and were 34% of the initial concentrations. The decrease in DOC concentration was higher than the decrease in TOC and ranged from 34 to 55% with the largest decline at 25°C and the lowest similarly at 9°C and 14°C (Fig 3). An increase in temperature of 10°C to 15°C produced an approximately 15% increase in TOC photodegradation and a 20% increase in DOC photodegradation.

Photodegradation of DOC, presented as a sum of rate constants k*_DOC→POC_ and *k*_*DOC→DIC*_ (transformation pathways 1 and 3 in Fig 2), ranged from 0.035 to 0.064 h^-1^ for 9°C and 25°C, respectively, and increased with increasing temperature (p < 0.001, r = 0.99) (Table 2). The sum of rate constants k*_DOC→POC_ and *k*_*DOC→DIC*_ almost doubled its value with a temperature increase of 15°C. This increase is similar to the twofold increase in photodegradation rate constants with each 10°C change assumed by Molot and Dillon [9] in a photodegradation study from the same location. Photo-oxidation of DOC to DIC, *k*_*DOC→DIC*_, dominated at lower temperatures, while at higher temperatures the transformation of DOC through the POC pathway, k*_DOC→POC_, was more significant (Table 2).

**Fig 4:**
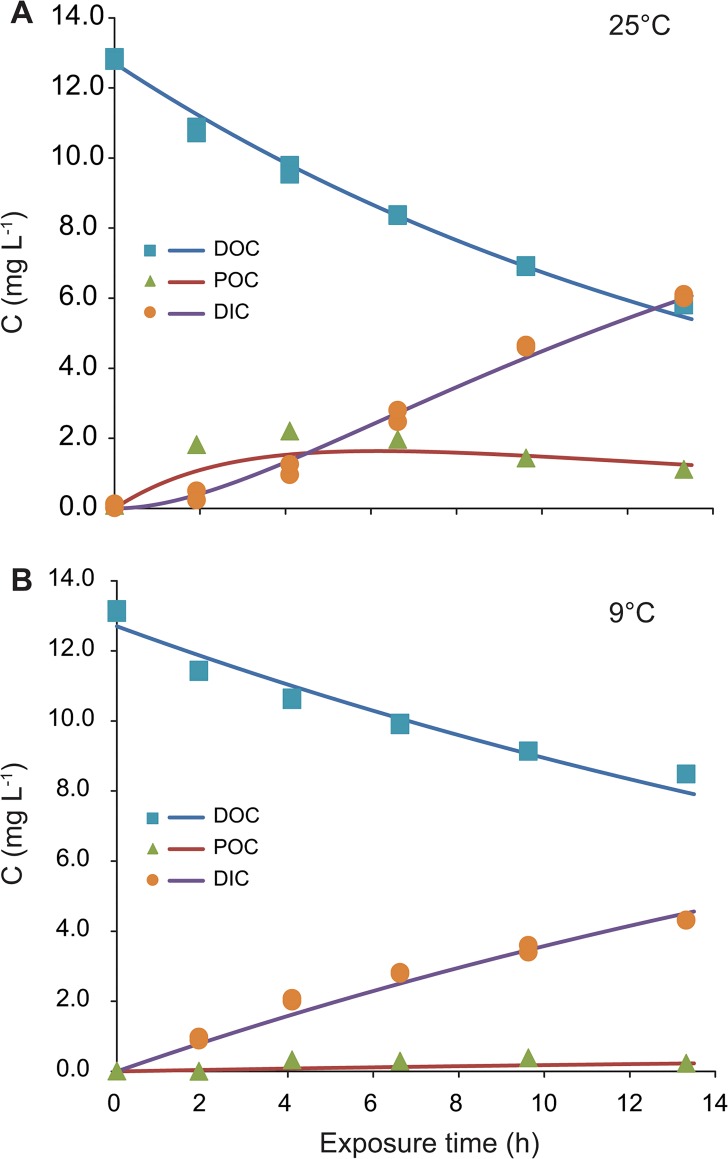
Changes in DOC, POC, and DIC concentrations during irradiation. **A–at 25°C; B–at 9°C.** Points represent measured data. Solid lines represent modeled trends.

The formation of POC during irradiation has been previously observed in several studies e.g. [14, 17, 26]. POC production calculated as a difference between TOC and DOC concentrations differed with temperature and was higher at higher temperatures (Fig 4). We observed a similar pattern in POC concentration in all experiments as the POC trend described in Porcal et al. [17]. Initially the POC concentration increased, reached its maximum and began to decrease. The maximum POC concentration reached at lower temperatures was approximately 20% of the maximum POC concentration of 2.2 mg L^-1^ at higher temperatures (Fig 4), it was 17% of the initial DOC concentration which corresponded to the range 15–30% of POC maximums observed by Porcal et al. [17] in experiments with samples from the same stream at room temperature. The POC concentration did not increased continuously at higher temperature (Fig 4A), but reached a maximum and then decreased similar to previous experiments [17]. This decline could be accounted for photochemical disaggregation of formed particles. One of the possible mechanisms of POC formation is the coagulation by inorganic forms of metals such as Al and Fe previously organically-bound and then released during irradiation as binding sites are destroyed [13, 14]. Changes in Al and Fe concentration during photochemical formation of particles in the same water samples due to the formation of insoluble hydrolysis products were documented by Porcal et al. [17]. Another possible mechanism of POC formation is the spontaneous assembly of dissolved organic matter into polymer gels [27].

Effects of temperature on coagulation and flocculation are driven by physical and chemical factors. During perikinetic coagulation, the first phase of coagulation, the transport of formed particles is driven by Brownian diffusion and temperature has a low effect. In the second phase, orthokinetic coagulation, flocs are formed and the flocculation rate can be affected by lower water viscosity and by water mixing at lower temperature [28]. Temperature affects the strength of flocs formed in this phase. Hanson and Cleasby [29] demonstrated that iron and aluminum flocs formed at 5°C were weaker than those formed at 20°C.

Chemical factors affect mainly solubility and speciation of metal ions. The minimum solubility of Fe(OH)_3(s)_ is higher at lower temperature and its optimum pH is shifted upwards with decreasing temperature [28]. Similar trends were confirmed for aluminum [30]. If, formation rates of particulate Fe and Al decrease with temperature, then we would expect adsorption of DOC to particulate Fe and Al to decrease which might account for the decrease in POC formation.

Van Benschoten and Edzwald [31] observed that the optimum pH for Al precipitation shifted from 4.6 at 25°C to 5.5 at 5°C and they also observed a shift in the isoelectric point of Al precipitates.

**Fig 5:**
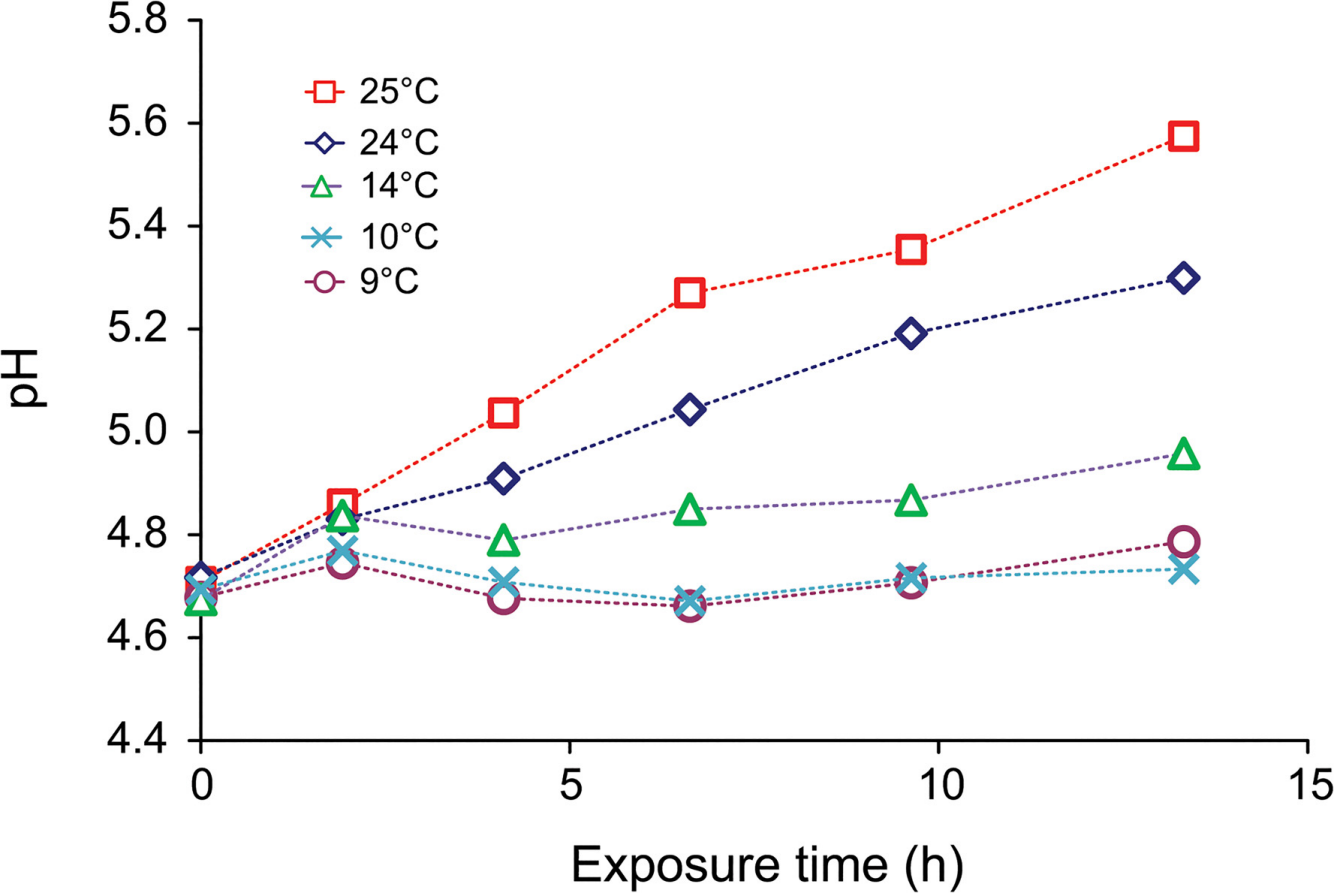
Changes in pH during irradiation experiments at different temperatures.

A strong temperature dependence of pH was observed in our experiments (Fig 5). One possible reason for the observed pH shift towards higher values was the change from DOM dominated buffer system to a carbonate buffer system, due to the degradation of strong and weak organic acid functional groups of DOM, microbial activity, and along with the decreasing solubility of carbon dioxide in water with increasing temperature. At 25°C, the final pH almost reached the theoretical pH of water in equilibrium with atmospheric CO_2_ of 5.65 [32]. At lower temperature the pH, which remained constant or only slightly increased during irradiation, was below optimum pH for coagulation of both iron and aluminum hydroxides. However, at higher temperatures, pH significantly increased during irradiation and was much closer to the coagulation optimum of iron and aluminum hydroxides than at lower temperature. The presence and photochemical release of previously organically bound iron and aluminum from DOM during irradiation was not measured in this study, but has been previously observed in irradiated samples from the same location [17].

The observed lower POC formation rate at low temperatures is in a good agreement with observations of Kang and Cleasby [28] who showed that low temperature slowed the growth of aggregates and enhanced the charge neutralizing ability of Fe coagulant.

The POC concentration did not increase consistently, but according to our experimental results, reached a maximum and then began to decrease. This decrease in POC concentration can be attributed to two processes: (i) sedimentation [33], and (ii) photochemical transformation of POC which can result in the production of lower molecular DOC or DIC [34]. The observed pattern in POC concentration was similar to the kinetics of an intermediate product in consecutive reactions. We simplified the photochemical processes of formation and degradation of POC into two “pseudo” consecutive reactions which can be described as formation of POC from DOC, k*_DOC→POC_, followed by decomposition of previously formed POC, *k*_*POC→DIC*_ (Eq 2). The latter step represents photo-oxidation of POC to DIC e.g. [35, 36, 37], or its sedimentation [33]. The possible POC photochemical transformation dissolution back to DOC was taken into account in the equilibrium rate constant k*_DOC→POC_. Sedimentation was negligible in the well mixed laboratory experiment but could play a significant role in the fate of photochemically formed POC in natural ecosystems.

The kinetic rate constant of POC degradation (*k*_*POC→DIC*_ in Eqs 2 and 5) “drives” the accumulation of POC in the system. The values of this rate constant ranged from 0.002 to 0.324 h^-1^ and increased with increasing temperature (p<0.001, r = 0.99) (Table 2).

The concentration of DIC was not measured directly, but was calculated from a TOC mass balance instead. DIC production was 34% of the initial TOC concentration at 9°C. At higher temperatures DIC production was higher (49% at 24°C and 46% at 25°C).

The increase in DIC concentration during irradiation is the result of two photo-oxidation processes. The first one is the photo-oxidation of DOC and the second one is the photo-oxidation of previously formed POC. Experimental results showed different trends in concentrations of DIC (Fig 4). At lower temperatures (9 to 14°C) the increase in DIC concentration was relatively constant, while at higher temperatures the increase in DIC concentration was slow at the beginning of the experiments, followed by a sharper increase.

Modeled DIC concentrations showed different ratios between two photo-oxidation processes responsible for DIC production. The production of DIC from DOC dominated at lower temperatures, while the production of DIC from POC was more significant at higher temperatures, as shown by the rate constants in Table 2. The photochemical oxidation of POC had a small effect on DIC concentration due to a lower POC formation rate at lower temperatures.

Since the samples were not sterilized by filtration through a 0.2 μm filter, but only through a 0.45 μm pore size filters, it is necessary to consider the possible role of an increase in microbial activity affecting DIC production due to microbial respiration and POC formation by microbial aggregation during five days of incubation mostly in experiments at higher temperatures. The higher degradation percentage of DOC at higher temperature may produce bioavailable low molecular weight organic compounds which, associated to the higher temperature, may stimulate microbial activity.

The decline in DOC concentration attributed to the microbial degradation of DOC was 3 ± 0.8% in dark control samples at higher temperatures. The contribution of microbial activity to DOC degradation in irradiated samples was not measured but can be estimated from the results of Köhler et al. [38], who measured microbial C uptake during photochemical degradation of water from a boreal forested watershed and concluded that integrated microbial uptake was 30% of TOC drop in stream water and photodegradation of TOC exceeded calculated microbial respiration by at least two-fold after 12 days of incubation.

The different pathways of DOC degradation observed at low and high temperature may be due, at least in part, to the combination of microbial activity and photodegradation at high temperature. We tried to estimate the possible effect of microbial processes in experiments at higher temperature. Measured concentrations of DIC, POC, and DOC were linearly amended of up to 30% for five days of incubation in respect to C mass balance. The correction factor of 30% was chosen according to Köhler et al. [38]. The kinetic rate constants for amended values representing only photochemical processes were modeled (for 25°C, *k*_*DOC→DIC*_ = 0, k*_DOC→POC_ = 0.0431, *k*_*POC→DIC*_ = 0.2955, R^2^ = 0.99). Obtained rate constants for amended photochemical processes were similar to the modeled values (Table 2). Thus, the possible microbial activity in experiments at higher temperature had small effect on observed pathways in DOM photochemical transformation. However, microbial DIC production from low molecular weight compounds is an important photochemically induced process. Its quantification was not determined in this study but was included in determination of DOC to DIC photochemical pathway.

The growth of bacteria and algae during five days of incubation in experiments at higher temperature would have resulted in an increase in POC, but equally increasing metal/carbon ratios in POC observed in similar experiments done with the same water at laboratory temperature [17] suggested that POC formation might be explained by continued formation of particulate metal coupled to declining availability of adsorbable DOC and/or photo-degradation of POC [39, 40] rather than microbial formation of POC which would have lower effect on metal/POC ratios.

## Conclusions

The results of our experiments and mathematical simulation of possible transformation processes showed significant dependence of photochemical transformation processes on temperature. There exist two possible photo-transformation pathways for transformation of DOC to DIC. The first pathway involves direct photo-oxidation and induced microbial mineralization of DOC to DIC and dominates at lower temperatures. The second pathway involves the formation of an intermediate, POC, and dominates at higher temperatures.

These observations are very important for correct transfer of laboratory experiments to natural conditions. Laboratory experiments are usually conducted under higher temperatures and their results correspond only to the short season of summer. However, the interpretation of results for low temperatures needs more attention. Results of experiments, usually conducted at temperatures higher than ambient temperatures in spring and fall, will need corresponding corrections.

Natural conditions in summer favor the DOC → POC pathway because water temperature and solar radiation are high whereas in spring when DOC fluxes are high [41] and more photoreactive [18] the DOC → DIC pathway may dominate which has implications for boreal C budgets. Photochemically formed POC can settle below the photolytic zone in summer before it is fully oxidized thereby limiting CO_2_ evasion to the atmosphere. Indeed, it may be a significant contribution to lake sediments.
